# Supplementary material for: Functional divergence of Capicua isoforms explains differential tissue vulnerability in neurological disease
Source: Genes Dev. Author manuscript; Available in PMC 2026 Jul 23. (PMC13390769; doi:10.1101/gad.353596.125)
Supplement: Supplementary file 1 [file NIHMS2155336-supplement-Supplementary_data.docx]

**Supplementary Tables**

**Supplemental Table S1. Overlapping differentially expressed genes between** ***Cic-S*****-KO and** ***Atxn1*****-KO cortex datasets. Related to Supplemental Figure S7.**

**Supplemental Table S2. Overlapping differentially expressed genes between** ***Cic-L*****-KO and** ***Atxn1*****-KO cortex datasets. Related to Supplemental Figure S7.**

**Supplemental Table S3. Overlapping differentially expressed genes between** ***Cic-S*****-KO and** ***Atxn1*****-KO cerebellum datasets. Related to Supplemental Figure S9.**

**Supplemental Table S4. Overlapping differentially expressed genes between** ***Cic-L*****-KO and** ***Atxn1*****-KO cerebellum datasets. Related to Supplemental Figure S9.**

**Supplementary Figures**

**Supplemental Figure S1. CIC-L, CIC-S, ATXN1, and ATXN1L expression in different brain regions.**

(A) Representative immunoblot of CIC-L, CIC-S, ATXN1, and ATXN1L in 12-week-old WT mouse brain lysates. CTX, cortex; HP, hippocampus; STR, striatum; CB, cerebellum; BS, brainstem. 

(B, C) Quantification of each protein's expression: CIC-L, CIC-S, ATXN1, and ATXN1L (B); Total CIC combining CIC-L and CIC-S (C). Protein levels were normalized to Vinculin (VCL), and values were normalized to the cortex value for each respective protein.

(D) Ratio of CIC-L to CIC-S at each region.

(E) Total CIC was normalized to ATXN1 for each region. Mean of each region compared to the normalized cortical expression of each protein. Data are mean ± SEM; n = 4 per region. One-way ANOVA with Dunnett’s multiple comparisons for (B-E); (*) p < 0.05, (**) p < 0.01, (***) p < 0.001, (****) p < 0.0001. 

**Supplemental Figure S2.** ***Cic-S*****-KO and** ***Cic-L*****-KO were generated by frameshift indels.**

(A) Schematic of *Cic-L*-KO and *Cic-S*-KO allele generation. *Cic-L-*KO was generated by a 1975bp deletion of the first coding exon of *Cic-L* (*Cic-L* exon2). The deletion is downstream of the start codon and leads to a frameshift of *Cic-L*. *Cic-S*-KO was generated by an indel that spans the *Cic-S* unique exon1 and the downstream intron. The deletion is downstream of the start codon and spans 50bp of the exon including the downstream start codon and 14bp of the intron with a one bp insertion. Overall, this leads to a frameshift of *Cic-S*. The start codon is marked with red and the downstream ATG is marked with black. The PCR primers for genotyping are marked as arrows. 

(B) Representative gel image of genotyping PCR reaction. *Cic-L* KO allele (383bp), *Cic-L* KO allele (403bp), *Cic-S* WT allele (633bp), and *Cic-S* KO allele (570bp).

(C) Sanger sequencing confirming a frameshift indel of *Cic-S*-KO (top), and *Cic-L*-KO (bottom) genomic DNA. The downstream ATG in *Cic-S* exon 1 is highlighted in yellow.

**Supplemental Figure S3.** ***Cic-L*****-KO shows motor deficits in the pole test and more time spent with partner during a social interaction assay.** 

(A) Fine motor behavior measured with pole test at 15-17 weeks of age. 

(B) Social behavior measured with the three-chamber assay at 12-15 weeks of age.
For (A) n = 17 (8 males, 9 females) WT, n = 8 *Cic-S*-KO (1 males, 7 females), and n =16 *Cic-L*-KO (9 males, 7 females) were analyzed with one-way ANOVA with Tukey’s multiple comparisons. For (B) n = 27 (12 males, 15 females) WT, n = 10 *Cic-S*-KO (3 males, 7 females), and n =14 *Cic-L*-KO (5 males, 9 females). Data are mean ± SEM. One-way ANOVA with Dunnett’s multiple comparisons for (A); Two-way ANOVA with Dunnett’s multiple comparisons for (B);  (***) p < 0.001, (****) p < 0.0001, (ns) p > 0.05. 

**Supplemental Figure S4.** ***Cic-S*****-KO shows greater number and magnitude of DEGs in the lungs compared to** ***Cic-L*****-KO DEGs.**

(A) Principal component analysis (PCA) of RNA-seq data from P6 lungs (WT, *Cic-S*-KO [SKO], *Cic-L*-KO [LKO]) using the top 500 most variable genes.

(B) Left: Venn diagram showing overlap of differentially expressed genes (DEGs; padj < 0.05 & |log2 fold change (log2FC)| >0.25) between *Cic-S*-KO and *Cic-L*-KO. Odds ratio (OR) and Fisher’s exact *p*-value are indicated. A total of 15473 genes were analyzed. Right: Scatterplot comparing log2FC of DEGs *Cic-S*-KO versus *Cic-L*-KO. Pearson correlation coefficient (r) and R² are shown. Overlapping DEGs marked in purple.

(C, D) Top gene ontology (GO) enrichment terms for biological processes of *Cic-S*-KO (C) and *Cic-L*-KO (D).

**Supplemental Figure S5.** ***Cic-S*****-KO DEGs show greater overlap with** ***Atxn1l*****-KO DEGs than** ***Cic-L*****-KO DEGs in lung.**

(A, B) Left: Venn diagrams showing overlap between DEGs in *Cic-S*-KO [SKO] (A) or *Cic-L*-KO [LKO] (B) and DEGs from *Atxn1l*-KO lungs reported in *Lee et al., 2011*. Odds ratio (OR) and Fisher’s exact test p-value are indicated. A total of 15473 genes were analyzed. Right: Scatterplot comparing log2FC of DEGs of *Cic-S*-KO (A) or *Cic-L*-KO (B) versus *Atxn1l-*KO. Pearson correlation coefficient (r) and R² are shown. Overlapping DEGs marked in blue (A) or red (B).

(C, D) Left: Venn diagrams showing overlap between DEGs in *Cic-S*-KO (A) or *Cic-L*-KO (B) and DEGs from *Atxn1*-KO lungs reported in *Lee et al., 2011*. OR and Fisher’s exact *p*-value are indicated. A total of 15473 genes were analyzed. Right: Scatterplot comparing log2FC of DEGs of *Cic-S*-KO (C) or *Cic-L*-KO (D) versus *Atxn1-*KO. Pearson correlation coefficient (r) and R² are shown. Overlapping DEGs marked in brown (C) or navy (D).

(E) Boxplot of *Etv-Mmp* genes. Differential expression statistics were obtained using DESeq2; (*) adjusted p < 0.05, (**) adjusted p < 0.01, (***), adjusted p < 0.001, (****) adjusted p < 0.0001. 

(F) Enrichment of GO of extracellular matrix assembly and disassembly terms of *Cic-S*-KO (Left) or *Cic-L*-KO (Right) gene set enrichment analysis (GSEA).

**Supplemental Figure S6.** ***Cic-L*****-KO shows greater number and magnitude of DEGs than** ***Cic-S*****-KO in the cortex.**

(A) Principal component analysis (PCA) of RNA-seq data from 12-week-old cortex of WT, *Cic-S*-KO [SKO], *Cic-L*-KO [LKO] of this study and 10-week-old cortex from *Atxn1*-KO [AKO] *(unpublished data)* and WT using the top 500 most variable genes.

(B) Left: Venn diagram showing overlap of DEGs between *Cic-S*-KO and *Cic-L*-KO. OR and Fisher’s exact *p*-value are indicated. A total of 15354 genes were analyzed. Right: Scatterplot comparing log2FC of DEGs *Cic-S*-KO versus *Cic-L*-KO. Pearson correlation coefficient (r) and R² are shown. Overlapping DEGs marked in purple.

(C, D) Top gene ontology (GO) terms of all biological processes (C) and neuro- or glia- related terms (D) of *Cic-S*-KO gene set enrichment analysis (GSEA).

(E, F) Top enriched GO terms of all biological process (E) and neuro- or glia- related -terms (F) of *Cic-L*-KO GSEA.

**Supplemental Figure S7.** ***Cic-L*****-KO DEGs show greater overlap with** ***Atxn1*****-KO DEGs than** ***Cic-S*****-KO DEGs in the cortex.**

(A, B) Left: Venn diagrams showing overlap between DEGs in *Cic-S*-KO [SKO] (A) or *Cic-L*-KO [LKO] (B) and DEGs (padj < 0.05 & |log2FC| >0.25) from *Atxn1*-KO cortex (*unpublished data)*. OR and Fisher’s exact *p*-value are indicated. A total of 15290 genes were analyzed. Right: Scatterplot comparing log2FC of DEGs of *Cic-S*-KO (A) or *Cic-L*-KO (B) versus *Atxn1-*KO. Pearson correlation coefficient (r) and R² are shown. Overlapping DEGs marked in brown (A) or navy (B).

(C, D) Top enriched gene ontology terms of biological process of the DEGs that overlap and change in the same direction between *Atxn1*-KO and *Cic-S*-KO (C), or *Atxn1*-KO and *Cic-L*-KO (D).

(E) Boxplot of *Etv1/4/5-Bace1*. Differential expression statistics were obtained using DESeq2; (****) adjusted p < 0.0001. 

**Supplemental Figure S8.** ***Cic-L*****-KO shows greater number and magnitude of DEGs in the cerebellum than** ***Cic-S*****-KO.**

(A) Principal component analysis (PCA) of RNA-seq data from 12-week-old cerebellum of WT, *Cic-S*-KO [SKO], *Cic-L*-KO [LKO] of this study and 10-week-old cerebellum *Atxn1*-KO [AKO] and WT (*unpublished data*) using the top 500 most variable genes.

(B) Left: Venn diagram showing overlap of DEGs between *Cic-S*-KO and *Cic-L*-KO. OR and Fisher’s exact *p*-value are indicated. A total of 15354 genes were analyzed. Right: Scatterplot comparing log2FC of DEGs *Cic-S*-KO versus *Cic-L*-KO. Pearson correlation coefficient (r) and R² are shown. Overlapping DEGs marked in purple.

(C, D) Top gene ontology (GO) terms of all biological process (C) and neuro- or glia- related terms (D) of *Cic-S*-KO gene set enrichment analysis (GSEA).

(E, F) Top enriched GO terms of all biological process (E) and neuro- or glia- related -terms (F) of *Cic-L*-KO GSEA.

**Supplemental Figure S9.** ***Cic-S*****-KO DEGs show greater overlap in the cerebellum with** ***Atxn1*****-KO DEGs than** ***Cic-L*****-KO DEGs.**

(A, B) Left: Venn diagrams showing overlap between DEGs in *Cic-S*-KO [SKO] (A) or *Cic-L*-KO [LKO] (B) and DEGs (padj < 0.05 & |log2FC| >0.25) from *Atxn1*-KO cerebellum (*unpublished data*). OR and Fisher’s exact *p*-value are indicated. A total of 15290 genes were analyzed. Right: Scatterplot comparing log2FC of DEGs of *Cic-S*-KO (A) or *Cic-L*-KO (B) versus *Atxn1-*KO. Pearson correlation coefficient (r) and R² are shown. Overlapping DEGs marked in brown (A) or navy (B).

(C) Boxplot of *Etv1/4/5-Bace1*. Differential expression statistics were obtained using DESeq2; (**) adjusted p < 0.01, (****) adjusted p < 0.0001. 

 

**Supplemental Figure S10. CIC, ATXN1, and ATXN1L expression in cortex and cerebellum of WT,** ***Cic-S*****-KO, and** ***Cic-L*****-KO mice.**

 (A, B) Representative immunoblots and quantification of CIC-L, CIC-S, ATXN1, and ATXN1L protein levels in cortex (A) and cerebellum (B) of 12-week-old WT, *Cic-S*-KO, and *Cic-L*-KO mice. Protein levels were normalized to VCL, and values were normalized to the WT. Data are mean ± SEM; n = 4 per region. One-way ANOVA with Dunnett’s multiple comparisons; (*) p < 0.05, (**) p < 0.01, (***) p < 0.001, (****) p < 0.0001, (ns) p>0.05.

**Supplemental Figure S11. CIC isoforms form different complexes with ATXN1 and ATXN1L in the WT cortex and cerebellum (related to Figure 5).**

(A-D) SEC elution profile of each protein of WT cortex and cerebellum. The percentage of each protein (mean ± SEM) in each fraction was determined from n = 3–4 independent cortical or cerebellar extracts. Protein abundance in each fraction was quantified by densitometry, with the signal in each fraction normalized to the total signal across all fractions for both regions. Two-way ANOVA with Šídák’s multiple comparisons; (*) p < 0.05, (**) p < 0.01, (***) p < 0.001, (****) p < 0.0001. 

**Supplemental Figure S12.** ***Cic, Atxn1,*** **and** ***Atxn1l*** **expression in mouse cerebellum at P60.**

**Supplemental Figure S13. CIC-L is more dependent on ATXN1 than CIC-S in all brain regions.**

(A-D) Representative immunoblots (top) and quantification of input (bottom) of ATXN1, ATXN1L and CIC expression in WT mice and *Atxn1*-KO mice of four different brain regions. Protein levels were normalized to VCL, then relative levels were normalized to WT expression.

(E) Binding preference of CIC-L/CIC-S in ATXN1 IP normalized to the ratio in input. CTX, cortex; HP, hippocampus; CB, cerebellum; BS, brainstem. Data are mean ± SEM; n = 4 per region. Welch’s t-test for (A-D); One-way ANOVA with Tukey’s multiple comparisons for (E). (*) p < 0.05, (**) p < 0.01, (ns) p > 0.05. 

**Supplementary Materials and Methods**

**Behavioral assays**

**Pole test**: Mice (15-17 weeks of age) were placed head-upward at the top of a vertical threaded metal pole. Time to descend to the base was recorded with a 60 s cutoff. If a mouse turned but fell before reaching the floor, the time to fall was recorded. Mice unable to turn downward were scored as 60 s.

**Three-chamber sociability assay**: Partner mice (matched for age, sex, and genetic background) were habituated to the three-chamber apparatus (length: 54 cm; width: 40 cm; height: 23 cm) divided into three chambers by removable partitions, for 1 h per day on two consecutive days prior to testing, while placed inside wire cups. On the test day, following habituation, experimental mice (12-15 weeks of age) were placed in the central chamber and allowed to freely explore for 10 min. A novel partner mouse was then placed inside a wire cup in either the left or right chamber, while a novel object was placed inside a wire cup in the opposite chamber. Placement of the partner mouse was randomized across trials. Experimental mice were allowed to explore for 10 min, and interaction time with each wire cup was manually scored.

**RNA extraction** 

Cortical and cerebellar tissues from 12-week-old mice, and lung tissues from P6 mice, were dissected, flash-frozen in liquid nitrogen, and stored at −80 °C until further processing. Total RNA was isolated using the miRNeasy Mini Kit (Qiagen, 217004) according to the manufacturer’s instructions. RNA concentration and purity were measured using a NanoDrop 1000 (Thermo Fisher).

**RNA sequencing and analysis**

RNA samples were submitted to Azenta Life Sciences for RNA integrity assessment, library preparation, and sequencing. Libraries were generated following polyA selection for mRNA and sequenced on Illumina NovaSeq or HiSeq platforms, yielding ~ 30 million 150-bp paired-end reads per sample.

For each sample, the raw reads from multiple lanes were merged, and then were aligned to the Mus musculus reference transcriptome (GRCm38.p6, GENCODE M18) using STAR v2.7 with "-quantMode GeneCounts" to generate raw read counts (Dobin et al. 2013).

Differential gene expression analysis was performed using DESeq2 v1.44.0 (Love et al. 2014). Principal component analysis (PCA) was performed on the top 500 most variable genes. Genes were considered differentially expressed if adjusted p < 0.05 and |log2 fold change| > 0.25.

Previously published lung P6 microarray data of *Atxn1*-KO and *Atxn1l-*KO were obtained from *Lee et al., 2011*. Differentially expressed genes (DEGs) reported in that dataset were used for comparison; the original publication defined DEGs using thresholds of p < 0.05 and |fold change| > 1.2. For *Atxn1*-KO RNA-seq data from cortex and cerebellum (*unpublished data*), read count data were processed with the same DESeq2 pipeline, and limma v3.60.6 (Ritchie et al. 2015) was used for batch correction prior to PCA.

Gene set enrichment analysis (GSEA) for Gene Ontology (GO) terms was conducted on all ranked genes using fgsea v1.30.0, with neuro/glia-related GO terms selected based on keywords including “neuron,” “neuro,” “nervous,” “synapse,” “axon,” “glia,” “glial,” “astrocyte,” “oligodendrocyte,” “microglia,” “myelin,” and “brain.” (Korotkevich et al. 2019) Overlapping differentially expressed genes were analyzed for GO term enrichment using clusterProfiler v4.12.6 (Xu et al. 2024), with the background universe defined as all genes detected in the RNA-seq dataset for the corresponding tissue.

**Supplementary References**

Dobin A, Davis CA, Schlesinger F, Drenkow J, Zaleski C, Jha S, Batut P, Chaisson M, Gingeras TR. 2013. STAR: ultrafast universal RNA-seq aligner. *Bioinformatics* **29**: 15-21.

Korotkevich G, Sukhov V, Sergushichev A. 2019. Fast gene set enrichment analysis. *bioRxiv*: 060012.

Love MI, Huber W, Anders S. 2014. Moderated estimation of fold change and dispersion for RNA-seq data with DESeq2. *Genome Biol* **15**: 550.

Ritchie ME, Phipson B, Wu D, Hu Y, Law CW, Shi W, Smyth GK. 2015. limma powers differential expression analyses for RNA-sequencing and microarray studies. *Nucleic Acids Res* **43**: e47.

Xu S, Hu E, Cai Y, Xie Z, Luo X, Zhan L, Tang W, Wang Q, Liu B, Wang R et al. 2024. Using clusterProfiler to characterize multiomics data. *Nat Protoc* **19**: 3292-3320.
